# Azilsartan Attenuates 3-Nitropropinoic Acid-Induced Neurotoxicity in Rats: The Role of IĸB/NF-ĸB and KEAP1/Nrf2 Signaling Pathways

**DOI:** 10.1007/s11064-023-04083-8

**Published:** 2024-01-07

**Authors:** Hend A. Hamouda, Rabab H. Sayed, Nihad I. Eid, Bahia M. El-Sayeh

**Affiliations:** 1 Department of Pharmacology and Toxicology, Faculty of Pharmacy, Cairo University, Kasr El Aini St., Cairo, 11562 Egypt; 2 School of Pharmacy, Newgiza University, Giza, Egypt

**Keywords:** Huntington’s disease, 3-nitropropionic acid, Azilsartan, NF-ĸB, Nrf2, Rat

## Abstract

Huntington’s disease (HD) is an autosomal-dominant neurodegenerative disorder characterized by motor, psychiatric and cognitive symptoms. Injection of 3-nitropropionic acid (3-NP) is a widely used experimental model for induction of HD. The current study aimed to inspect the potential neuroprotective properties of azilsartan (Azil), an angiotensin II type 1 receptor blocker (ATR1), in 3-NP-induced striatal neurotoxicity in rats. Rats were randomly allocated into five groups and treated for 14 days as follows: group I received normal saline; group II received Azil (10 mg/kg, p.o.); group III received 3-NP (10 mg/kg, i.p); group IV and V received Azil (5 or 10 mg/kg, p.o, respectively) 1 h prior to 3-NP injection. Both doses of Azil markedly attenuated motor and behavioural dysfunction as well as striatal histopathological alterations caused by 3-NP. In addition, Azil balanced striatal neurotransmitters levels as evidenced by the increase of striatal gamma-aminobutyric acid content and the decrease of glutamate content. Azil also amended neuroinflammation and oxidative stress via modulating IĸB/NF-ĸB and KEAP1/Nrf2 downstream signalling pathways, as well as reducing iNOS and COX2 levels. Moreover, Azil demonstrated an anti-apoptotic activity by reducing caspase-3 level and BAX/BCL2 ratio. In conclusion, the present study reveals the neuroprotective potential of Azil in 3-NP-induced behavioural, histopathological and biochemical changes in rats. These findings might be attributed to inhibition of ATR1/NF-κB signalling, modulation of Nrf2/KEAP1 signalling, anti-inflammatory, anti-oxidant and anti-apoptotic properties.

**Graphical Abstract:**

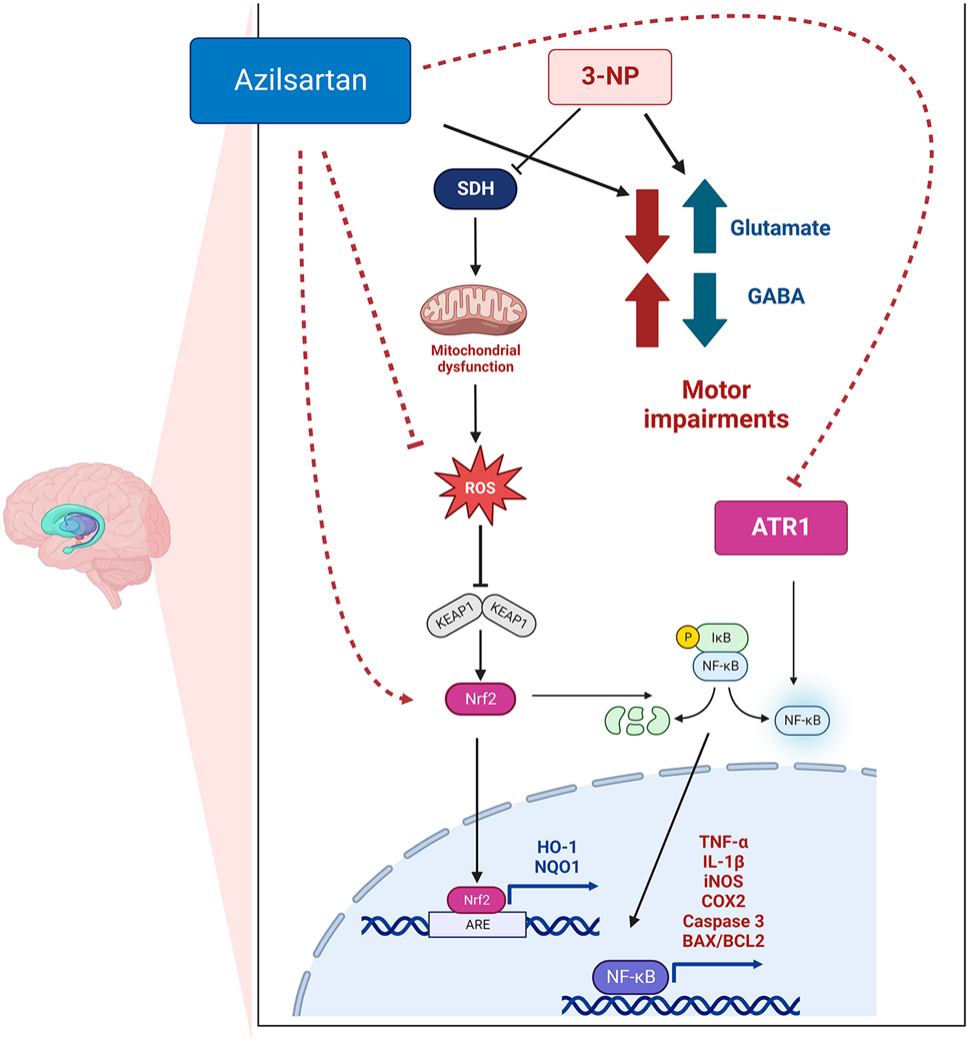

## Introduction

Huntington’s disease (HD) is an autosomal-dominant neurodegenerative disorder caused by the expansion of the CAG trinucleotide repeat in the Huntingtin (Htt) gene leading to the production of a mutant huntingtin protein (mHtt) [1–3]. Clinical symptoms of HD include motor, psychiatric and cognitive [4–6]. 3-Nitropropionic acid (3-NP) is a mitochondrial toxin that can effectively produce the symptoms of HD in animals and serve as an experimental model of HD [7, 8]. 3-NP can cross the blood–brain barrier (BBB) and inhibit succinate dehydrogenase (SDH) enzyme irreversibly, blocking the electron transport chain and leading to ATP depletion, up-surge in reactive oxygen species (ROS) and depletion of endogenous antioxidants thus producing mitochondrial dysfunction and neuronal apoptosis [9–11].

Nuclear factor-kappa B (NF-κB) is a transcription factor principally involved in immune, inflammatory, and stress responses [12]. NF-κB is also involved in neuronal injury, making it a potential therapeutic target for managing neurodegenerative disorders [13]. Besides its role in the regulation of the transcription of the genes responsible for inflammation, NF-κB regulates the transcription of genes implicated in the apoptotic process [14]. In the basal status, NF-κB is maintained in an inactive form in the cytosol by binding to a repressive protein, an inhibitor of nuclear factor kappa B (IκB), to form an inactive protein complex that inhibits the nuclear translocation of NF-κB [15]. Numerous pro-inflammatory stimuli can activate NF-κB, mainly through inhibitor of κB kinase (IKK)-based phosphorylation and degradation of IκB proteins [16], where such inflammatory stimuli as well as, cellular stresses result in IκB phosphorylation by IKK leading to the activation and nuclear translocation of NF-ĸB [17, 18]. Then, NF-κB activates the transcription of growth factors, chemokines, cytokines and pro-apoptotic factors-encoding genes [19, 20].

The nuclear factor erythroid 2-related factor 2 (Nrf2) is considered a vital controller of redox homeostasis that coordinates the endogenous antioxidant cellular response [21, 22]. In normal circumstances, Nrf2 levels are preserved low in the cytoplasm by binding to Kelch-like ECH-associated protein 1 (KEAP1). Exposure to ROS disrupts the KEAP1-Nrf2 complex with consequent release of Nrf2 which translocates into the nucleus to bind to antioxidant response elements (ARE) promoting the transcription of numerous antioxidative stress-related genes including haem oxygenase-1 (HO-1) and NAD(P)H: quinone oxidoreductase-1 (NQO-1) [23, 24]. The Nrf2 signalling pathway is also involved in the inhibition of NF-ĸB as well as, its downstream inflammatory cytokines [25]. The key role of Nrf2 in hindering oxidative stress in HD has been suggested because Nrf2 knocked out mice proved to be more susceptible to striatal lesions induced by 3-NP [26].

Azilsartan (Azil) is an angiotensin II type 1 receptor blocker (ARB) that is used as an antihypertensive drug [27]. Azil has been shown to possess anti-inflammatory, anti-oxidant and anti-apoptotic effects that are associated with neuroprotection via blocking brain angiotensin II type 1 receptors (AT1R) [28–31]. Besides, the downstream signalling pathway of renin–angiotensin–aldosterone system (RAS) has been associated with NF-kB activation [32]. Thus, the current study aimed to investigate the potential neuroprotective effect of Azil against 3-NP-induced neurotoxicity in rats via assessing various behavioural, biochemical, and histopathological parameters. Moreover, we also investigated the effect of Azil on the interplay between IĸB/NF-ĸB and KEAP1/Nrf2 signalling pathways.

## Material and Methods

### Animals

Adult male Wistar rats (200–250 g) were purchased from the animal colony of the Faculty of Pharmacy, Cairo University, Egypt. Rats were kept under the proper conditions of suitable humidity (60–70%), ventilation (10–20 changes/h), temperature (25 ± 2 °C), and constant 12/12 h light/dark cycle with free access to a standard rodent chow diet and water. The study adheres to the Guide for Care and Use of Laboratory Animals published by the US National Institutes of Health (NIH Publication No. 85-23, revised 2011) and was approved by the Ethics Committee for Animal Experimentation at the Faculty of Pharmacy, Cairo University (Permit Number: PT 2503).

### Drugs and Chemicals

3-NP was bought from Sigma-Aldrich Chemical Co. (St. Louis, MO, USA), while Azil was procured from Rameda Pharmaceutical (Egypt). 3-NP and Azil were dissolved in normal saline (0.9%) for intraperitoneal (i.p.) injection and oral (p.o.) administration, respectively. All other chemicals used in the study were from the top grade available commercially.

### Experimental Design

**Fig. 1:**
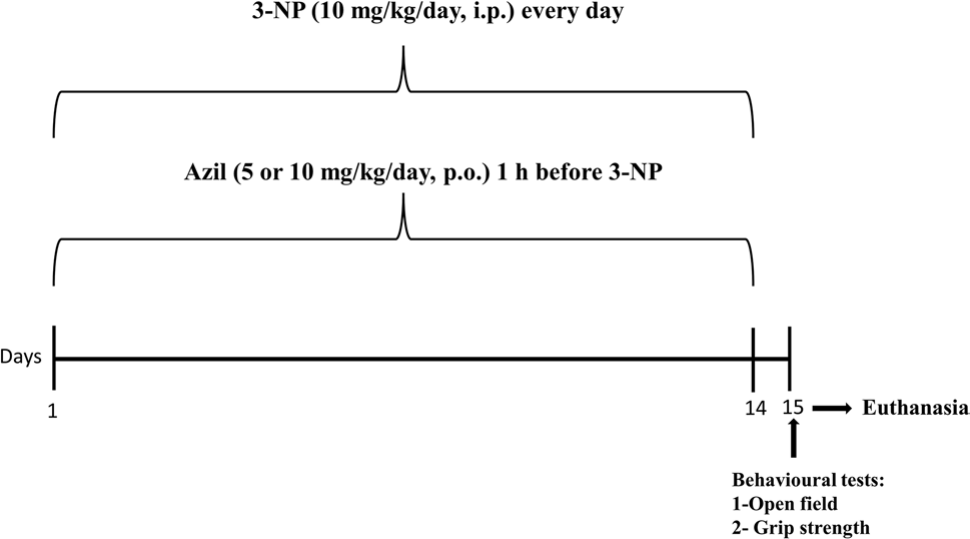
Timeline of the experimental design. 3-NP 3-nitropropionic acid, Azil azilsartan

As depicted in Fig. 1, rats were randomly assigned into 5 groups, 9 rats per group and treated for 14 days as follows: Group I (Control) received i.p. injection of normal saline and served as the normal control group; Group II (Azil) received Azil (10 mg/kg, p.o.); Group III (3-NP) received i.p. injection of 3-NP (10 mg/kg/day, i.p.) [33]; Group IV (3-NP + Azil 5) received azil (5 mg/kg, p.o.) [34] 1 h before 3-NP injection and Group V (3-NP + Azil 10) received Azil (10 mg/kg, p.o.) [35] 1 h before 3-NP injection.

### Behavioral Assessments

Twenty-four hours after the last 3-NP injection, rats were subjected to behavioural tests namely; open field and grip strength tests.

#### Open Field Test

Open field test was done to assess behavioural responses like spontaneous locomotor activity and exploratory behaviour of rats [36]. The test was done in an 80 × 80 cm wooden box with a height of 40 cm. The floor of the box was divided into 16 squares with white lines separating them. The test was done in a quiet room under white light. Each rat was placed in the centre of the open field box gently and the locomotor activity was recorded for 3 min. The box was cleaned with 10% isopropyl alcohol and dried carefully for each animal to avoid any disturbing substances left by the previous animal. The following parameters were recorded for each animal during the 3 min assessment period [37, 38].

- The latency time: time passed until the animal decides to move from the starting point (the central area) measured in seconds.
- Ambulation frequency: the number of squares crossed over by the animals.
- Rearing frequency: number of times the animal stood, stretched on its hind limbs with or without the support of the forelimb.

#### Grip Strength Test

The grip strength of rats was evaluated by a rat grip strength meter (Model 47200, Ugo Basile, Comerio, Italy) [39]. Rats were carefully placed over a base plate forward-facing a triangle bar. When the rat gripped the bar by its forelimbs, it was gently dragged by its tail horizontally backward away from the triangle bar until its forelimbs are released. The maximum pulling force (g) was recorded when the animal lost its grip on the grasping bar (when its front paws grasping the bar were released). For each rat, the average of three values was recorded.

### Brain Processing

After the behavioural assessments, rats were sacrificed by decapitation under anaesthesia with thiopental. Brains were quickly removed and rinsed with ice-cold saline. Two sets of rats were designated for each group: one for histological examination and the other for biochemical parameters. In the first set of samples (n = 3), brains were fixed in 10% (v/v) buffered formalin for 72 h to perform staining with haematoxylin and eosin (H&E) for the histopathological examination. In the second set of samples (n = 6), right striatum was properly separated and homogenized in ice-cold saline to prepare a 10% homogenate for the assessment of malondialdehyde (MDA), succinate dehydrogenase (SDH) by colorimetric technique, glutamate, gamma-aminobutyric acid (GABA), haem oxygenase-1 (HO-1), NAD(P)H: quinone oxidoreductase-1, tumor necrosis factor-alpha (TNF-α), interleukin-1 beta (IL-1β), cyclooxygenase-2 (COX-2), inducible nitric oxide synthases (iNOS), caspase-3, Bcl-2-associated X protein (BAX) and B-cell lymphoma 2 (BCL2) by using rat ELISA kits. Left striatum of the second set was used for assessment of IκB, NF-κB p65, KEAP1 and Nrf2 by Western blot analysis as well as AT1R using RT-PCR.

### Biochemical Parameters

#### Colorimetric Assay

MDA content and SDH activity were determined in the striatal homogenate colorimetrically using a specific kit (Biodiagnostic, Egypt, Cat. No. MD25 28 and Biovision, USA, Cat. No. K660-100), respectively.

### Enzyme-Linked Immunoassay (ELISA) Technique

In ice-cold phosphate-buffered saline, striata were homogenized to yield 10% homogenates and the content of each parameter in the striatum was determined using the matching rat-specific commercial kits according to the manufacturer’s instructions. TNF-α, BAX, and BCL2 were assessed using Cusabio (Wuhan, China) ELISA kits (Cat. No. CSB-E11987r, CSB-EL002573RA and CSB-E08854r, respectively). Moreover, MyBiosource (San Diego, CA, USA) ELISA kits were used for determination of glutamate, GABA, IL-1β, NQO1, HO-1, COX-2, caspase-3 and iNOS (Cat. No. MBS756400, MBS045103, MBS825017, MBS7606601, MBS 764989, MBS 266603, MBS7244630 and MBS 263618, respectively).

#### Western Blot Technique

Using RIPA lysis buffer, the separated striatal tissues were lysed and their protein content was determined using the Bradford protein assay kit (Thermo Fisher Scientific Inc., MA, USA) according to the method of Bradford [40]. After protein quantification of striata, 10 μg of total protein was separated by Sodium Dodecyl Sulfate PolyAcrylamide Gel Electrophoresis gel (SDS-PAGE) and transferred onto polyvinylidene difluoride membranes (Pierce, Rockford, IL, USA). To block the non-specific binding sites, membranes were soaked in tris-buffered saline with Tween 20 (TEST) buffer and 3% bovine serum albumin (BSA) at room temperature for 1 h. Afterward, membranes were incubated overnight at 4 °C with the primary antibodies directed against IκB (Cat. no. MA5-15132), NF-κB p65 (Cat. no. 436700), KEAP1 (Cat. no. PA5-99434), Nrf2 (Cat. no. PA5-27882) and β-actin (Cat. no. MA5-15,739). The blots were incubated with horseradish peroxidase-conjugated secondary antibody (Dianova, Hamburg, Germany) at 37 °C and left for 1 h after washing them many times. Protein bands were obtained by an enhanced chemiluminescence substrate reaction (Amersham Biosciences, Arlington Heights, IL, USA). Using densitometric analysis utilizing a scanning laser densitometer (Biomed Instrument, Inc., CA, USA), the corresponding intensities of the protein bands were measured. The results were expressed as arbitrary units relative to the intensity of the corresponding β-actin bands.

### Quantitative Real Time-PCR (qRT-PCR)

Striatal AT1R gene expression was detected by RT-PCR. SV total RNA extraction kit (Invitrogen, CA, USA) was used to extract RNA from the striatal tissues. The extracted RNA was reverse transcribed into cDNA using RT-PCR kit (Thermo Fisher Scientific, MA, USA) according to the manufacturer’s instructions. The primer sequences were as follows: AT1R, F: GCACACTGGCAATGTAATGC, R: GTTGAACAGAACAAGTGACC and ß-Actin, F: CCCATCTATGAGGGTTACGC, R: TTTAATGTCACGCACGATTTC. The PCR reactions were set up in 50 µl reaction mixtures, which contained 25 μl SYBR green mix, 0.5 μl cDNA, 2 μl primer pair mix (5 pmol/μl each primer) and 22.5 μl RNAse free water. PCR program was set up as follows: 95 °C for 10 min, followed by 45 cycles of 15 s (denaturation) and 1 min at 60 °C (annealing/extension). The target gene’s relative expression was estimated using the 2 ^− ΔΔCT^ formula. β-Actin was used as a housekeeping gene to normalize the mRNA levels of the target gene.

### Histopathological Examination

Brains were washed and fixed in 10% (v\v) buffered formalin for 72 h. Then, samples were processed to be embedded in paraffin with the preparation of 3 μm sections. Tissue sections were stained by hematoxylin and eosin (H&E) as a general staining method and inspected microscopically by light microscope (magnification × 200 and × 400). Images were captured and processed using Adobe Photoshop (version 8.0).

Immunohistochemical staining of glial fibrillary acidic protein (GFAP) was performed using a rat monoclonal antibody (Santa Cruz Biotechnology, TX, USA). All procedures were performed according to the manufacturer’s instructions. The extent of positive immunostaining in five random non-overlapping fields per tissue section was calculated as the area percentage of expression using cellSens Dimension software (Olympus software).

### Statistical Analysis

The results were analysed by one-way ANOVA followed by Tukey's multiple comparisons tests, except for the GFAP area%, which were analysed using Kruskal–Wallis ANOVA followed by Dunn's multiple comparison test. All results were expressed as mean ± S.D. Statistical analysis was achieved using GraphPad Prism software (version 6). A probability level of < 0.05 was accepted in all statistical tests as statistically significant.

## Results

Noteworthy, no significant difference was detected between normal control rats and those that received Azil alone in all assessed parameters.

### Effect of Azil (5 or 10 mg/kg) on 3-NP-Induced Behavioral Abnormalities

**Fig. 2:**
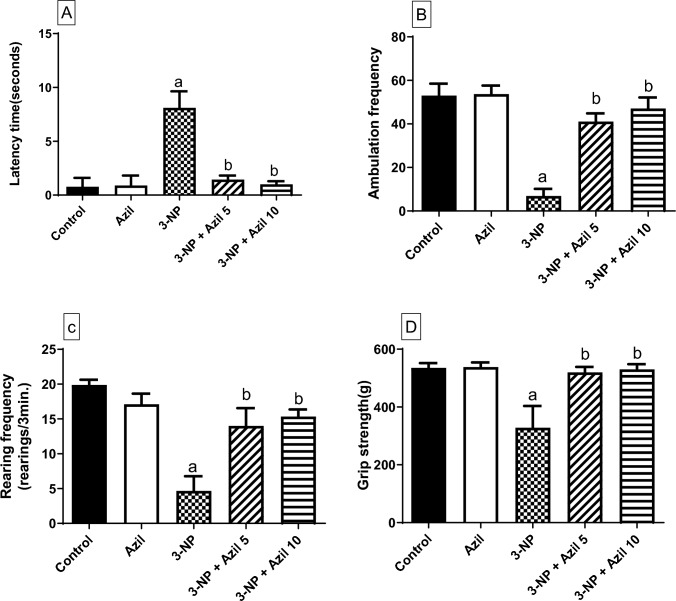
Effect of Azil (5 or 10 mg/kg) on 3-NP-induced behavioral abnormalities. **A** Latency time, **B** Ambulation frequency, **C** Rearing frequency and **D** Grip strength. Each bar represents mean ± S.D. (n = 9). Statistical analysis was carried out by one-way ANOVA followed by Tukey’s multiple comparisons test. a: significantly different from the control group at P ≤ 0.05. b: significantly different from 3-NP-treated group at P ≤ 0.05. Azil azilsartan, 3-NP 3-nitropropionic acid

As shown in Fig. 2, 3-NP-intoxicated animals displayed behavioral and motor deteriorations, as evidenced by the open field (latency time, ambulation frequency and rearing frequency) and grip strength tests. 3-NP injected rats showed a significant increase of the latency time (about 10 -folds that of the control group), F (4, 40) = 76.45, P < 0.0001. Moreover, 3-NP group rats exhibited a marked decrease in ambulation frequency (12.99% of control group), rearing frequency (23.46% of control group), and grip strength (61.38% of control group), F (4, 40) = 21.66, 32.04, and 22.42 (P < 0.0001), respectively. Treatment with Azil (5 mg/kg) produced a significant decrease in the latency time (17.80% of 3-NP group) along with an increase in ambulation frequency, rearing frequency, and grip strength (about 6-, 3- and 1.6- folds that of the 3-NP group, respectively). Similarly, administration of Azil (10 mg/kg) ameliorated the aforementioned behavioral changes as evidenced by a decrease of latency time (12.32% of 3-NP group), meanwhile the ambulation, rearing frequencies as well as the grip strength were markedly increased (about 7-, 3- and 1.7-folds that of 3-NP-treated rats, respectively).

### Effect of Azil (5 or 10 mg/kg) on 3-NP-Induced Changes in Striatal Neurotransmitters

**Fig. 3:**
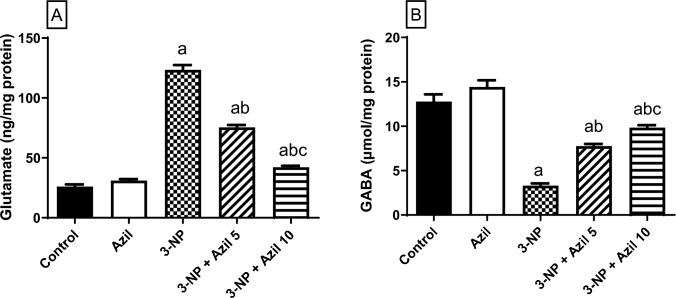
Effect of Azil (5 or 10 mg/kg) on 3-NP-induced changes in striatal neurotransmitters **A** glutamate and **B** GABA. Each bar represents mean ± S.D. (n = 6). Statistical analysis was carried out by one-way ANOVA followed by Tukey’s multiple comparisons test. a: significantly different from the control group at P ≤ 0.05. b: significantly different from 3-NP-treated group at P ≤ 0.05. c: significantly different from Azil (5 mg/kg)-treated group at P ≤ 0.05. Azil Azilsartan, 3-NP 3-nitropropionic acid, GABA γ-amino butyric acid

As depicted in Fig. 3, 3-NP-treated rats displayed GABA and glutamate striatal imbalance as shown by the significant rise in glutamate level (about fivefolds that of the control group) and the prominent reduction in GABA level (25.88% of the control rats), F (4, 25) = 628.3 and 96.37, P < 0.0001, respectively. Conversely, Azil (5 mg/kg) administration succeeded to decrease the glutamate level (61.10% of 3-NP group) and replenishing the GABA level (about 2.3-folds that of 3-NP-treated animals). Meanwhile, administration of Azil (10 mg/kg) showed more significant decrease in the elevated glutamate level (34.14% of 3-NP group) and also raised the GABA level to reach (about threefolds that of the 3-NP-treated rats).

### Effect of Azil (5 or 10 mg/kg) on 3-NP-Induced Alterations of Striatal AT1R Expression and NF-ĸB Signalling Pathway Parameters

**Fig. 4:**
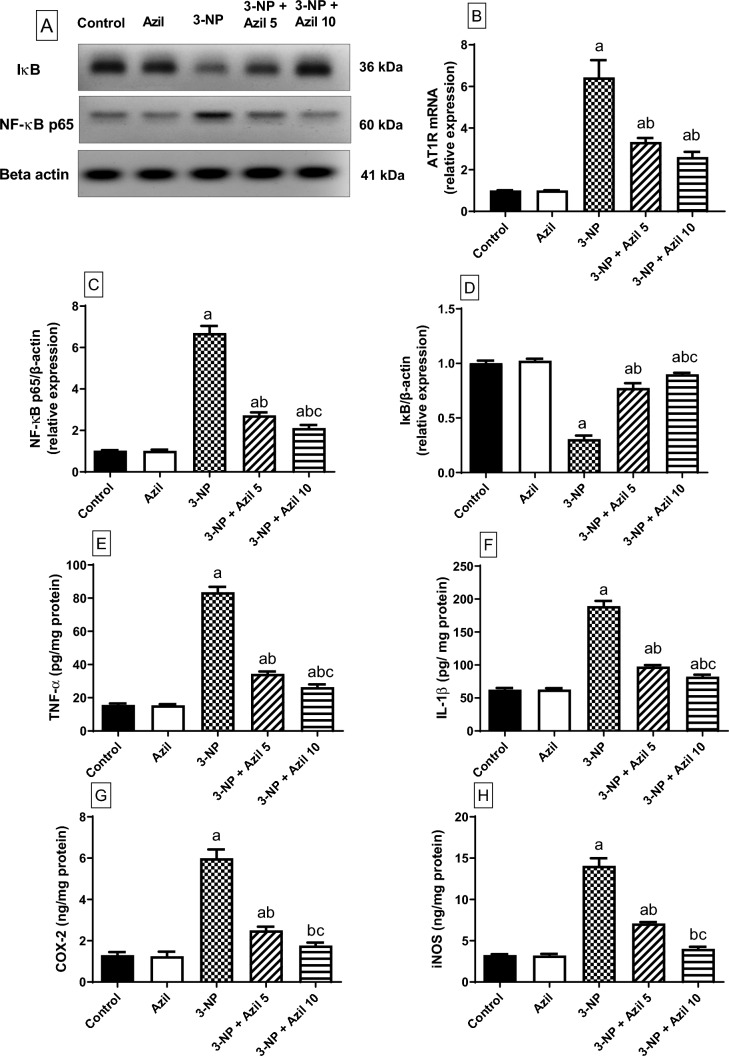
Effect of azilsartan (5 or 10 mg/kg) on 3-NP-induced changes in striatal NF-κB signalling pathway parameters. **A** Densitometric analysis of the Western blots, **B** ATR1 mRNA expression, **C** NF-κB p65 expression, **D** IκB expression, **E** TNF-α content, **F** IL-1β content, **G** COX-2 content, and **H** iNOS content**.** Each bar represents mean ± S.D. (n = 6). Statistical analysis was carried by one-way ANOVA followed by Tukey’s multiple comparisons test. a: significantly different from the control group at P ≤ 0.05. b: significantly different from 3-NP-treated group at P ≤ 0.05. c: significantly different from Azil (5 mg/kg)-treated group at P ≤ 0.05. Azil Azilsartan, 3-NP 3-nitropropionic acid, AT1R angiotensin II receptor type 1, IκB inhibitor of kappa-B, NF-κB nuclear factor kappa-b P65, TNF-α tumor necrosis factor-alpha, IL-1β interleukin-1 beta, COX-2 cyclooxygenase-2 and iNOS inducible nitric oxide synthases

As presented in Fig. 4A and B, the expression of AT1R and NF-κB p65 were significantly elevated in 3-NP-treated rats (about 6.5-folds that of the control rats, respectively), F (4, 10) = 56.86 and F (4, 25) = 118.6, respectively. In contrast, 3-NP intoxication caused a down-regulation of IκB expression to (30.57% that of the control rats), F (4, 25) = 281, (P < 0.0001) (Fig. 4C). However, 3-NP-induced increment in AT1R and NF-κB p65 expression were hampered by Azil (5 mg/kg) (51.84% and 40.79% of the 3-NP-treated rats’ values, respectively). Moreover, treatment with Azil (5 mg/kg) mitigated the depletion of the IκB expression (about 2.5-folds the 3-NP-treated rats’ values). The striatal levels of AT1R and NF-κB p65 expression were markedly reduced by Azil (10 mg/kg) administration (40.62% and 31.59% of the 3-NP-treated rats’ values, respectively). Meanwhile, treatment with Azil (10 mg/kg) significantly raised the IκB expression (about threefolds that of 3-NP-treated rats).

### Effect of Azil (5 or 10 mg/kg) on 3-NP-Induced Alterations of Striatal Inflammatory Parameters

As presented in Fig. 4D–G, 3-NP intoxication increased the striatal levels of TNF-α, IL-1β, COX2 and iNOS (about 5.3-, 3-, 4.6- and 4.3-folds that of the control rats, respectively), F (4, 25) = 511.1, 383.8, 175.3 and 104.4, respectively. However, 3-NP-induced increment in TNF-α, IL-1β, COX-2 and iNOS was hampered by Azil (5 mg/kg) (41.12%, 51.53%, 41.66% and 50.34% of the 3-NP-treated rats’ values, respectively). Meanwhile, the striatal levels of TNF-α, IL-1β, COX2 and iNOS were markedly reduced by Azil (10 mg/kg) administration (31.60%, 43.49%, 29.45% and 28.55% of the 3-NP-treated rats’ values, respectively).

### Effect of Azil (5 or 10 mg/kg) on 3-NP-Induced Changes in Striatal Nrf2 Signalling Pathway Parameters

**Fig. 5:**
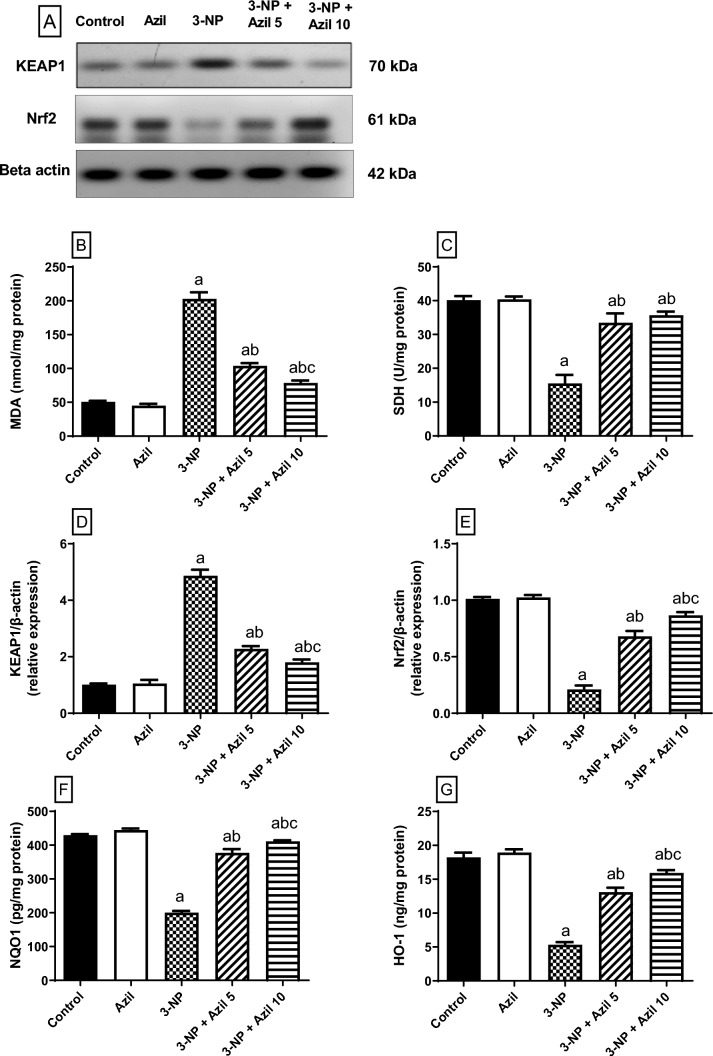
Effect of Azil (5 or 10 mg/kg) on 3-NP-induced changes in striatal Nrf2 signalling pathway parameters.** A** Densitometric analysis of the Western blots, **B** MDA content, **C** SDH activity, **D** KEAP1 expression, **E** Nrf2 expression, **F** NQO-1 content, and **G** HO-1 content. Each bar represents mean ± S.D. (n = 6). Statistical analysis was carried by one-way ANOVA followed by Tukey’s multiple comparisons test. a: significantly different from the normal control group at P ≤ 0.05. b: significantly different from 3-NP-treated group at P ≤ 0.05. c: significantly different from Azil (5 mg/kg)-treated group at P ≤ 0.05. Azil Azilsartan, 3-NP 3-nitropropionic acid, MDA malondialdehyde, SDH succinate dehydrogenase, KEAP1 Kelch-like ECH-associated protein -1, Nrf2 nuclear factor erythroid 2-related factor 2, NQO-1: NAD(P)H quinone oxidoreductase-1and HO-1: heme oxygenase-1

Data in Fig. 5 shows that 3-NP injection produced a significant upsurge in striatal MDA content and KEAP1 expression (about 4- and 4.8-folds that of the control group, respectively), F (4, 25) = 388.1 and 400.8 P < 0.0001, respectively. However, 3-NP depleted striatal SDH activity, NQO-1, and HO-1 contents as well as Nrf2 expression (38.62%, 46.70% and 29.25%, and 20.73% that of the control group, respectively), F (4, 25) = 92.34, 591.3, 138.8, 253.7, P < 0.0001, respectively. Interestingly, Azil (5 mg/kg/day) administration hampered 3-NP-induced elevation in MDA content and KEAP1 expression (51.20% and 46.90% that of 3-NP group values, respectively). Moreover, striatal SDH, NQO-1, and HO-1 as well as Nrf2 expression were remarkably up-regulated in Azil 5 mg-treated rats (about 2.2-, 2-, 2.5- and 3.2-folds that of 3-NP group values, respectively). Meanwhile, administration of Azil (10 mg/kg) decreased MDA striatal content and KEAP1 expression (38.74% and 36.98% that of the 3-NP-treated rats, respectively). Moreover, striatal contents of SDH, NQO1 and HO1 as well as Nrf2 expression was significantly elevated in Azil 10 mg-treated rats (2.3-, 2-, 3- and 4-folds, that of 3-NP group values, respectively).

### Effect of Azil (5 or 10 mg/kg) on 3-NP-Induced Changes in Striatal Apoptosis

**Fig. 6:**
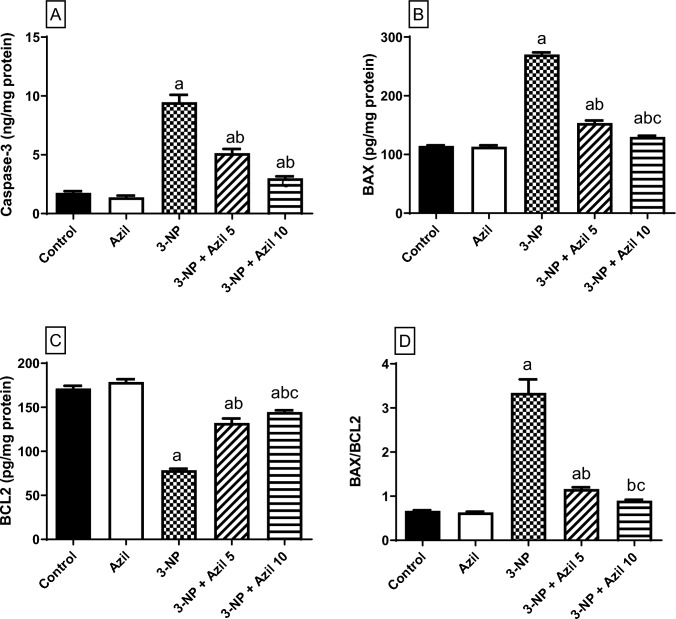
Effect of azilsartan (5 or 10 mg/kg) on 3-NP-induced changes in striatal apoptosis. **A** caspase-3 content, **B** BAX content, **C** BCL2 content, and **D** BAX/BCL2 ratio. Each bar represents mean ± S.D. (n = 6). Statistical analysis was carried out by one-way ANOVA followed by Tukey’s multiple comparisons test. a: significantly different from the normal control group at P ≤ 0.05. b: significantly different from 3-NP-treated group at P ≤ 0.05, c: significantly different from Azil (5 mg/kg)-treated group at P ≤ 0.05. Azil Azilsartan, 3-NP 3-nitropropionic acid, BAX Bcl-2-associated X protein and BCL2 B-cell lymphoma 2

Figure 6 reveals that 3-NP markedly increased striatal caspase-3 and BAX content (about 5.4-and 2.4-folds that of the control group, respectively), F (4, 25) = 215.3 and 574.3, respectively, while increasing Bcl2 content (45.83% of the control), F (4, 25) = 258.2, P < 0.0001, producing a marked elevation in the BAX/BCL2 ratio (about 5-folds that of the control), F (4, 25) = 360.6. On the other hand, treatment with Azil (5 mg/kg) succeeded in reversing the increase in caspase-3, BAX and BAX/BCL2 ratio (54.39%, 56.82% and 34.78% that of the 3-NP-treated group, respectively). Moreover, Azil (5 mg/kg)-treated rats exhibited a 1.7-folds increase in BCL2 as compared to 3-NP-treated rats. Meanwhile, Azil (10 mg/kg)-treated rats demonstrated a decline in striatal caspase-3, BAX as well as, BAX/BCL2 (31.68%, 48.02% and 26.87% that of 3-NP-treated rats, respectively). Furthermore, Azil (10 mg/kg)-treated rats exhibited a 1.8-folds increase in BCL2 as compared to 3-NP-treated animals.

### Effect of Azil (5 or 10 mg/kg) on 3-NP-Induced Histopathological Changes

**Fig. 7:**
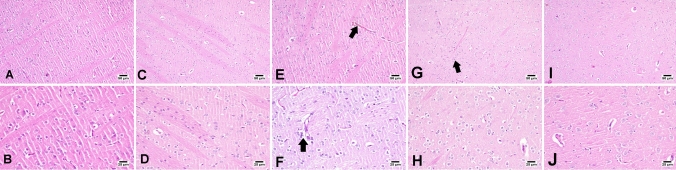
Effect of Azil (5 or 10 mg/kg) on 3-NP-Induced Histopathological Changes. Photomicrographs of sections **A** and **B** show normal histological structure of the striatum of rats receiving saline (control group). Photomicrographs of sections **C** and **D** show the normal histological structure of the striatum of rats receiving Azil alone. Photomicrograph of sections **E** of 3-NP treated rats shows congested blood vessels (arrow) with focal gliosis and **F** neuronal and perivascular lymphocytic infiltration and edema (arrow). Photomicrograph of sections **G** and **H** of Azil 5 mg-treated rat shows few degenerating cells (arrow) and **H** mild neuronal edema. Photomicrograph of section **I** and **J** of Azil 10 mg-treated rat shows apparently normal striatum with no histopathological changes

Microscopic examination of the striatum region of the brain from the control group (Fig. 7 and B) and Azil group (Fig. 7C and D) showed normal striatal structure. However, brain sections of the 3-NP-treated group (Fig. 7E and F) revealed congested blood vessels with focal gliosis, numerous dark degenerating neurons, and numerous dilated vessels with perivascular and neural edema. On the other hand, brain sections from 3-NP rats treated with Azil (5 mg/kg) (Fig. 7G and H) showed few degenerating cells and mild neuronal edema. Besides, brain sections from 3-NP rats treated with Azil (10 mg/kg) (Fig. 7I and J) showed normal structure with no histopathological changes.

### Effect of Azil (5 or 10 mg/kg) on 3-NP-Induced Changes in GFAP Expression

**Fig. 8:**
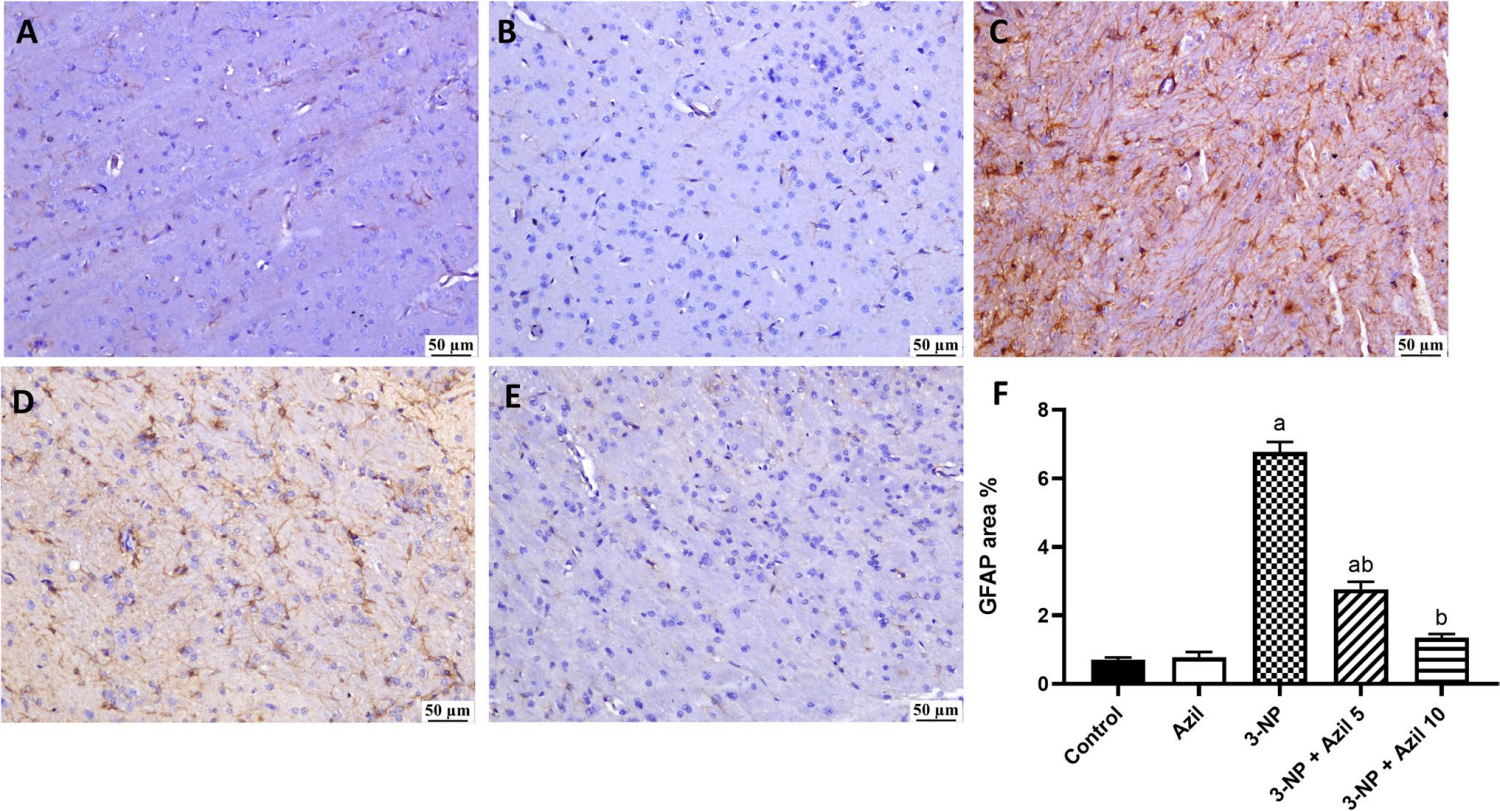
Effect of Azil (5 or 10 mg/kg) on 3-NP-induced changes in GFAP expression. Control (**A**) and Azil (**B**) groups exhibited mild GFAP expression in the striatum region. In contrast. 3-NP group (**C**) exhibited significant increase in GFAP expression. Lower expression levels were detected in 3-NP + Azil 5 (**D**) and 3-NP + Azil 10 (**E**) groups. **F**: GFAP immunostaining area %. Each bar represents mean ± S.D. (n = 3). Statistical analysis was carried out by Kruskal–Wallis ANOVA followed by Dunn’s multiple comparison test. a: significantly different from the normal control group at P ≤ 0.05. b: significantly different from 3-NP-treated group at P ≤ 0.05, c: significantly different from Azil (5 mg/kg)-treated group at P ≤ 0.05. Azil Azilsartan, 3-NP 3-nitropropionic acid, GFAP glial fibrillary acidic protein

Control group showed mild GFAP expression in the striatum region (Fig. 8A). Similarly, Azil group exhibited mild GFAP positive staining (Fig. 8B). In contrast, 3-NP-treated rats exhibited significant increase in GFAP expression (Fig. 8C). However, 3-NP rats treated with Azil (5 mg/kg) showed less expression levels of GFAP (Fig. 8D). Similarly, 3-NP rats treated with Azil (10 mg/kg) demonstrated less GFAP expression (Fig. 8E).

## Discussion

The current study verified the potential neuroprotective effect of both doses of Azil (5 or 10 mg/kg) in 3-NP-induced neurotoxicity in rats. This notion is evidenced by (i) an improvement in 3-NP-induced motor dysfunction; (ii) the increase of striatal GABA content and the decrease of glutamate content; (iii) the mitigation of 3-NP-induced striatal inflammation evidenced by the inhibition of NF-κB with its downstream inflammatory pathway, (v) the alleviation of 3-NP-induced striatal oxidative stress evidenced by the activation of Nrf2 with its downstream anti-oxidant pathway (vi) the improvement of 3-NP-induced striatal apoptosis and (vii) the improvement in striatal histopathological changes mediated by 3-NP.

Injection of 3-NP to rats is considered as a well-established experimental model of HD. When given systemically, 3-NP can easily cross the BBB [41] and cause bilateral striata lesions [42] and therefore, mimics the symptoms and neuropathology of HD in humans [43–45]. Herein, 3-NP intoxication resulted in diminished locomotor activity and loss of grip strength as proved by the results of the open field and grip strength tests, indicating motor impairment and striatal neurodegeneration, like that established in the late stages in HD patients [46]. These results go in line with previous studies demonstrating similar pattern of behavioural and motor abnormalities after 3-NP injection [47–49]. 3-NP-induced neurodegeneration was further confirmed by the histopathological examination, where the striatum of 3-NP-treated rats presented numerous degenerating neurons and congested blood vessels with focal gliosis, confirmed by the significant increase in GFAP immunoexpression. GFAP is an acidic protein that exclusively exists in the astrocytes and plays a significant role in astrogliosis in central nervous systems injuries and neurodegeneration [50]. Such neuronal damage is similar to what reported by prior studies [49, 51, 52]. However, Azil administration 1 h before 3-NP successfully diminished the striatal degeneration as well as motor impairment induced by 3-NP. These were evidenced by the marked improvement of locomotor activity and grip strength of those rats as compared to 3-NP-treated rats. Moreover, Azil prevented 3-NP-induced histopathological changes, where sections from stria of treated rats were apparently normal with no pathological changes and demonstrated less striatal GFAP expression. The neuroprotective effect of Azil was previously reported against rotenone-induced rat model of Parkinson’s disease [34], cerebral ischemia rat model [52] and aluminium chloride-induced neurobehavioral and pathological changes in rats [53].

The involvement of striatal neurotransmitters, GABA and glutamate, in HD pathogenesis as well as in 3-NP-induced experimental neurotoxic model is well reported [54]. 3-NP-induced neurotoxicity is known to be selective to the GABAergic neurons of the striatum [22]. Moreover, it is well recognized that 3-NP-triggered neurotoxicity incorporates glutamate-related excitotoxicity [55], where 3-NP was reported to produce an excessive release of glutamate in experimental rat striatal tissues [51]. Taken together, 3-NP-induced neurotoxicity could partially be due to the imbalance between the excitatory glutamate and the inhibitory GABA neurotransmitter in the striatum. This complies with the outcomes of the present study, where 3-NP-treated rats demonstrated elevation of striatal glutamate content along with decline of GABA content in the striatum. In contrast, Azil succeeded to restore the neurotransmitters balance, further confirming its neuroprotective potential in 3-NP-induced neurotoxicity.

The dysregulation of RAS was reported to be implicated in several neurodegenerative disorders [56, 57]. The mRNA expression of AT1R is reported to be elevated in all brain areas in a transgenic model of HD [57]. Similarly, in the current study, the expression of AT1R was significantly increased in 3-NP-treated rats. NF-ĸB is a critical player in the pathogenesis of several neurodegenerative disorders [58, 59]. The downstream signalling of AT1R is proven to lead to the activation of NF-κB [60]. In addition, it was reported that NF-κB activity is significantly up-regulated in the striatum of wild-type animals following 3-NP intoxication [33, 61] and in cultured mHtt-expressing cells [62]. Therefore, blocking NF-κB signal pathway is believed to be a potential target that could partially diminish 3-NP-induced striatal toxicity. In the resting state, NF-ĸB is held in the cytoplasm through association with IĸB proteins. Stimuli that trigger the stimulation of the IKK, result in phosphorylation and degradation of IĸB proteins with the consequent translocation of NF-ĸB to the nucleus, where it indorses the transcription of target genes encoding pro-inflammatory cytokines such as IL-1β, TNF-α and IL-6 [63]. It is also described that the elevation in TNF-α can also induce NF-κB activation [64, 65]. The results of the present study confirmed the implication of neuroinflammation initiated by AT1R/NF-ĸB pathway activation in 3-NP-induced neurotoxicity. 3-NP-treated rats showed elevated expression of AT1R and NF-ĸB p65 along with diminished IκB expression in the striatum with the consequent elevation of downstream pro-inflammatory cytokines including TNF-α and IL-1β. These results are in line with previous ones demonstrating significant elevation of NF-ĸB and pro-inflammatory mediators in 3-NP-treated rats [66, 67]. However, Azil showed an anti-inflammatory activity via reducing the AT1R and NF-ĸB p65 expression and the TNF-α and IL-1β levels along with increasing the expression of IκB. In line, Azil was previously reported to inhibit NF-κB and its downstream inflammatory mediators in renal ischemia reperfusion rat model [30].

The up-regulated expression of COX-2 has been considered as a significant cause of the neurotoxicity accompanied with inflammation [68, 69]. Herein, 3-NP-treated rats demonstrated significant elevation in striatal COX-2 content, which is in line with previous results [59, 70]. However, treatment with Azil lead to suppression of striatal COX-2 level as compared with 3-NP group. In agreement, Azil was previously reported to down-regulate COX-2 in a rat model of experimental periodontitis [28] and endometriosis [71]. Since it is well identified that COX-2 expression is mediated by NF-ĸB activation [72], the suppressing effect of Azil on COX-2 level could be explained by its NF-ĸB inhibitory action and this effect also contributes to its anti-inflammatory properties.

Additionally, iNOS is an enzyme which is induced during inflammatory conditions and it has been reported to be involved in neurodegenerative diseases [73]. iNOS can binds to COX-2 and boost its activity [74]. Thus, increased iNOS level, as reported herein in 3-NP-treated rats, leads to rise in COX-2 activation. In parallel, increased iNOS striatal content was previously reported in 3-NP-induced neurotoxicity in rats [75]. However, treatment with Azil successfully down-regulated iNOS expression compared to 3-NP-treated rats. Therefore, iNOS inhibition by Azil may have further reduced COX-2 level as well as inflammatory deterioration in striatum. A previous study reported that Azil suppress iNOS expression in lipopolysaccharide-activated microglia [76].

Mitochondrial dysfunction is a major hallmark of HD in human and it is considered as one of the key features of 3-NP-induced HD in animal models [49, 77]. 3-NP irreversibly inhibits SDH enzyme which is a crucial enzyme in the electron transport chain, Krebs cycle and superoxide control [78]. Inhibition of SDH leads to impeding energy production [7, 79, 80], triggers mitochondrial dysfunction [47], and causes excessive oxidative stress response that consequently leads to neuronal injury [22]. Since striatal neurons are extremely sensitive to derangement in energy metabolism, such mitochondrial dysfunction increase the susceptibility of striatum to acute intoxication with mitochondrial toxins such as 3-NP in both experimental and clinical studies [80]. Herein, 3-NP administration was associated with inhibition of striatal SDH activity in rats which go in line with previous studies [49, 67, 80], an effect that was reversed by Azil restoring mitochondrial activity. These findings comply with previous ones demonstrating that treatment with Azil significantly decreased the activity of SDH in high-fat diet (HFD)-induced sarcopenic obesity in rats [81]. Azil has been also reported to attenuate oxidative injury in brain endothelial cells via regulating mitochondrial activity [29]. A previous study also showed that pretreatment with Azil restores mitochondrial viability as well as the activities of mitochondrial complexes in cerebral ischemia in rats [52].

Further, the 3-NP-triggered oxidative stress was evident in the current study by the increased MDA striatal level signifying elevated lipid peroxidation. However, treatment with Azil reduced MDA level in striatum of 3-NP-treated rats, demonstrating its anti-oxidant properties that could contribute to its neuroprotective potential. Azil was previously reported to ameliorate oxidative stress in ethanol-induced gastric ulcers in rats [82] and cerebral ischemia reperfusion at model [83].

The oxidative stress injurious effects are usually offset by Nrf2, which is a major controller of the antioxidant-response that up-regulates genes of phase II detoxifying enzymes, such as HO-1 and NQO-1 [84]. These enzymes guard against the ROS-induced body damage [85]. Ubiquitination and degradation of Nrf2 in the cytoplasm is mediated by KEAP1, so that activation of Nrf2 depends on KEAP1 dissociation [86]. Upon activation, Nrf2 translocates into the nucleus and binds to antioxidant response elements (ARE) promoting the transcription of many antioxidant-related genes [87]. The vital role of Nrf2 signalling in hampering oxidative stress in HD animal models has been reported [26, 88]. In the present study, striatum of 3-NP-treated rats showed suppression of Nrf2 expression and up-regulation of KEAP1 expression along with decrease in level of Nrf2-targeted anti-oxidant enzymes (HO-1 and NOQ-1). This finding is consistent with a preceding study reporting that Nrf2 expression was significantly diminished in the striatum of 3-NP rats [75]. Contrariwise, Azil administration was associated with a significant decrease of the repressor KEAP1 and increase in Nrf2 expression compared to 3-NP treated group. This increase in Nrf2 expression was accompanied by an increase in HO-1 and NOQ-1 levels. In line with those results, it was reported that Azil diminishes lipopolysaccharide-induced acute lung injury in mice via increasing the expression of both Nrf2 and HO-1 [89]. These results suggest that activation of Nrf2 signalling pathway may be a possible mechanism by which Azil can alleviate oxidative stress.

Nrf2 and NF-κB signalling pathways are engaged in functional cross-talk [90]. For instance, the absenteeism of Nrf2 can aggravate NF-κB stimulation which leads to amplified cytokine production, while NF-κB can regulate the transcription and activity of Nrf2 [90] and thus can affect the antioxidant machinery [72]. Both of those transcription factors can form a crossing point to control the expression of many target proteins [91, 92]. Therefore, the neuroprotective effect showed by Azil in the current study could be, in part, attributed to the cross talk between NF-ĸB and Nrf2 signalling pathways.

Additionally, our results displayed that 3-NP injection potentiated striatal apoptosis as indicated by the upsurge in the level of caspase-3 and BAX/BCL2 ratio, which is in line with former studies [22, 47]. The potentiated apoptotic pathway associated with 3-NP administration could be attributed to generation of superoxide radicals following SDH inhibition [93]. In contrast, administration of Azil controlled the level of these apoptotic proteins in the striatum indicating a significant anti-apoptotic effect. The anti-apoptotic potential of Azil was also previously reported in renal ischemia reperfusion injury in rat [30]

Finally, our study is limited by the idea that the blood vessels congestion in brain striatum elicited by 3-NP could have facilitated the access of Azil into brain striatum. Additionally, Azil might be protective against the diffusion of 3-NP into the striatum of the rat brains analysed in the present study. These assumptions need to be investigated in future studies.

In conclusion, the current study demonstrates the potential neuroprotective effects of Azil, an ARB, against 3-NP-induced behavioural, histopathological, and biochemical changes in the striatum of rats. These findings might be attributed to inhibition of ATR1/NF-κB signalling, modulation of Nrf2/KEAP1 signalling, anti-inflammatory, anti-oxidant, and anti-apoptotic properties.

## Data Availability

Enquiries about data availability should be directed to the authors.

## References

[1] MacDonald ME, Ambrose CM, Duyao MP, Myers RH, Lin C, Srinidhi L (1993). A novel gene containing a trinucleotide repeat that is expanded and unstable on Huntington’s disease chromosomes. Cell.

[2] von Hörsten S, Schmitt I, Nguyen HP, Holzmann C, Schmidt T, Walther T (2003). Transgenic rat model of Huntington’s disease. Hum Mol Genet.

[3] Roos RAC (2010). Huntington’s disease: a clinical review. Orphanet J Rare Dis.

[4] Walker FO (2007). Huntington’s disease. Lancet.

[5] Finkbeiner S (2011). Huntington’s disease. Cold Spring Harb Perspect Biol.

[6] Heemskerk AW, Roos RAC (2011). Dysphagia in Huntington’s disease: a review. Dysphagia.

[7] Kumar P, Kumar A (2009). Neuroprotective effect of cyclosporine and FK506 against 3-nitropropionic acid induced cognitive dysfunction and glutathione redox in rat: possible role of nitric oxide. Neurosci Res.

[8] Silva-Palacios A, Ostolga-Chavarría M, Buelna-Chontal M, Garibay C, Hernández-Reséndiz S, Roldán FJ (2017). 3-NP-induced Huntington’s-like disease impairs Nrf2 activation without loss of cardiac function in aged rats. Exp Gerontol.

[9] Jadiswami C, Megha HM, Dhadde SB, Durg S, Potadar PP, Thippeswamy BS (2014). Piroxicam attenuates 3-nitropropionic acid-induced brain oxidative stress and behavioral alteration in mice. Toxicol Mech Methods.

[10] Dhadde SB, Nagakannan P, Roopesh M, Anand Kumar SR, Thippeswamy BS, Veerapur VP (2016). Effect of embelin against 3-nitropropionic acid-induced Huntington’s disease in rats. Biomed Pharmacother.

[11] Sharma P, Kumar M, Bansal N (2021). Ellagic acid prevents 3-nitropropionic acid induced symptoms of Huntington’s disease. Naunyn Schmiedebergs Arch Pharmacol.

[12] Kowalczyk A, Kołodziejczyk M, Gorąca A (2015). Nuclear factor κB inhibitor BAY 11–7082 suppresses oxidative stress induced by endothelin-1 (ET-1) in rat kidney. Adv Hyg Exp Med.

[13] Gupta S, Sharma B (2014). Pharmacological benefit of I(1)-imidazoline receptors activation and nuclear factor kappa-B (NF-κB) modulation in experimental Huntington’s disease. Brain Res Bull.

[14] Tsuruta D (2009). NF-kappaB links keratinocytes and lymphocytes in the pathogenesis of psoriasis. Recent Pat Inflamm Allergy Drug Discov.

[15] Huang TT, Kudo N, Yoshida M, Miyamoto S (2000). A nuclear export signal in the N-terminal regulatory domain of IκBα controls cytoplasmic localization of inactive NF-κB/IκBα complexes. Proc Natl Acad Sci.

[16] Chen L, Ruan Y, Wang X, Min L, Shen Z, Sun Y (2014). BAY 11–7082, a nuclear factor-κB inhibitor, induces apoptosis and S phase arrest in gastric cancer cells. J Gastroenterol.

[17] Zanotto-Filho A, Delgado-Cañedo A, Schröder R, Becker M, Klamt F, Moreira JCF (2010). The pharmacological NFκB inhibitors BAY117082 and MG132 induce cell arrest and apoptosis in leukemia cells through ROS-mitochondria pathway activation. Cancer Lett.

[18] Krishnan N, Bencze G, Cohen P, Tonks NK (2013). The anti-inflammatory compound BAY 11–7082 is a potent inhibitor of protein tyrosine phosphatases. FEBS J.

[19] Ghosh S, Karin M (2002). Missing pieces in the NF-κB puzzle. Cell.

[20] Kanarek N, Ben-Neriah Y (2012). Regulation of NF-κB by ubiquitination and degradation of the IκBs. Immunol Rev.

[21] Kulasekaran G, Ganapasam S (2015). Neuroprotective efficacy of naringin on 3-nitropropionic acid-induced mitochondrial dysfunction through the modulation of Nrf2 signaling pathway in PC12 cells. Mol Cell Biochem.

[22] Habib MZ, Tadros MG, Abd-Alkhalek HA, Mohamad MI, Eid DM, Hassan FE (2022). Harmine prevents 3-nitropropionic acid-induced neurotoxicity in rats via enhancing NRF2-mediated signaling: Involvement of p21 and AMPK. Eur J Pharmacol.

[23] Sykiotis GP, Habeos IG, Samuelson AV, Bohmann D (2011). The role of the antioxidant and longevity-promoting Nrf2 pathway in metabolic regulation. Curr Opin Clin Nutr Metab Care.

[24] Moretti D, Tambone S, Cerretani M, Fezzardi P, Missineo A, Sherman LT (2021). NRF2 activation by reversible KEAP1 binding induces the antioxidant response in primary neurons and astrocytes of a Huntington’s disease mouse model. Free Radic Biol Med.

[25] Mémet S (2006). NF-κB functions in the nervous system: from development to disease. Biochem Pharmacol.

[26] Shih AY, Imbeault S, Barakauskas V, Erb H, Jiang L, Li P (2005). Induction of the Nrf2-driven antioxidant response confers neuroprotection during mitochondrial stress in vivo. J Biol Chem.

[27] White WB, Weber MA, Sica D, Bakris GL, Perez A, Cao C (2011). Effects of the angiotensin receptor blocker azilsartan medoxomil versus olmesartan and valsartan on ambulatory and clinic blood pressure in patients with stages 1 and 2 hypertension. Hypertension.

[28] De Araújo AA, Varela H, De Brito GAC, De Medeiros CACX, De Araújo LS, Do Nascimento JHO (2014). Azilsartan increases levels of IL-10, down-regulates MMP-2, MMP-9, RANKL/RANK, Cathepsin K and up-regulates OPG in an experimental periodontitis model. PLoS ONE.

[29] Liu H, Mao P, Wang J, Wang T, Xie CH (2016). Azilsartan, an angiotensin II type 1 receptor blocker, attenuates tert-butyl hydroperoxide-induced endothelial cell injury through inhibition of mitochondrial dysfunction and anti-inflammatory activity. Neurochem Int.

[30] Alaaeldin R, Bakkar SM, Mohyeldin RH, Ali FEM, Abdel-Maqsoud NMR, Fathy M (2023). Azilsartan modulates HMGB1/NF-κB/p38/ERK1/2/JNK and apoptosis pathways during renal ischemia reperfusion injury. Cells.

[31] Raheem NM, Mohammed Ali MN (2023). Azilsartan suppresses the antiapoptotic biomarker and pro-inflammatory cytokines in rat model of cisplatin-induced retinal and optic nerve toxicity. Hum Exp Toxicol.

[32] Nagai N, Izumi-Nagai K, Oike Y, Koto T, Satofuka S, Ozawa Y (2007). Suppression of diabetes-induced retinal inflammation by blocking the angiotensin II type 1 receptor or its downstream nuclear factor-κB pathway. Invest Ophthalmol Vis Sci.

[33] Kumar P, Kalonia H, Kumar A (2011). Role of LOX/COX pathways in 3-nitropropionic acid-induced Huntington’s Disease-like symptoms in rats: protective effect of licofelone. Br J Pharmacol.

[34] Gao Q, Ou Z, Jiang T, Tian YY, Zhou JS, Wu L (2017). Azilsartan ameliorates apoptosis of dopaminergic neurons and rescues characteristic parkinsonian behaviors in a rat model of Parkinson’s disease. Oncotarget.

[35] Garg S, Arya DS (2018). A9641 novel angiotensin II receptor blocker azilsartan protects myocardium from ischemic reperfusion injury in experimental rats through peroxisome proliferator-activated receptor- γ activation. J Hypertens.

[36] Walsh RN, Cummins RA (1976). The open-field test: a critical review. Psychol Bull.

[37] El-Shamarka MES, El-Sahar AE, Saad MA, Assaf N, Sayed RH (2022). Inosine attenuates 3-nitropropionic acid-induced Huntington’s disease-like symptoms in rats via the activation of the A2AR/BDNF/TrKB/ERK/CREB signaling pathway. Life Sci.

[38] Farid HA, Sayed RH, El-Shamarka MES, Abdel-Salam OME, El Sayed NS (2023). PI3K/AKT signaling activation by roflumilast ameliorates rotenone-induced Parkinson’s disease in rats. Inflammopharmacology.

[39] Huang GJ, Edwards A, Tsai CY, Lee YS, Peng L, Era T (2014). Ectopic cerebellar cell migration causes maldevelopment of Purkinje cells and abnormal motor behaviour in Cxcr4 null mice. PLoS ONE.

[40] Bradford MM (1976). A rapid and sensitive method for the quantitation of microgram quantities of protein utilizing the principle of protein-dye binding. Anal Biochem.

[41] Stavrovskaya AV, Voronkov DN, Yamshchikova NG, Ol’shanskiy AS, Khudoerkov RM, Illarioshkin SN (2017). Experience of experimental modelling of Huntington’s disease. Hum Physiol.

[42] Danduga RCSR, Dondapati SR, Kola PK, Grace L, Tadigiri RVB, Kanakaraju VK (2018). Neuroprotective activity of tetramethylpyrazine against 3-nitropropionic acid induced Huntington’s disease-like symptoms in rats. Biomed Pharmacother.

[43] Alberch J, Pérez-Navarro E, Canals JM (2009). Animal models of Huntington’s disease. Encycl Neurosci.

[44] Túnez I, Tasset I, La Cruz VP, De SA (2010). 3-nitropropionic acid as a tool to study the mechanisms involved in Huntington’s disease: past, present and future. Molecules.

[45] Hariharan A, Shetty S, Shirole T, Jagtap AG (2014). Potential of protease inhibitor in 3-nitropropionic acid induced Huntington’s disease like symptoms: Mitochondrial dysfunction and neurodegeneration. Neurotoxicology.

[46] Suganya SN, Sumathi T (2017). Effect of rutin against a mitochondrial toxin, 3-nitropropionicacid induced biochemical, behavioral and histological alterations-a pilot study on Huntington’s disease model in rats. Metab Brain Dis.

[47] El-Abhar H, Abd El Fattah MA, Wadie W, El-Tanbouly DM (2018). Cilostazol disrupts TLR-4, Akt/GSK-3β/CREB, and IL-6/JAK-2/STAT-3/SOCS-3 crosstalk in a rat model of Huntington’s disease. PLoS ONE.

[48] Elbaz EM, Helmy HS, El-Sahar AE, Saad MA, Sayed RH (2019). Lercanidipine boosts the efficacy of mesenchymal stem cell therapy in 3-NP-induced Huntington’s disease model rats via modulation of the calcium/calcineurin/NFATc4 and Wnt/β-catenin signalling pathways. Neurochem Int.

[49] Sayed NH, Fathy N, Kortam MA, Rabie MA, Mohamed AF, Kamel AS (2020). Vildagliptin attenuates Huntington’s disease through activation of GLP-1 receptor/PI3K/Akt/BDNF pathway in 3-nitropropionic acid rat model. Neurotherapeutics.

[50] Yang Z, Wang KKW (2015). Glial fibrillary acidic protein: from intermediate filament assembly and gliosis to neurobiomarker. Trends Neurosci.

[51] Shalaby HN, El-Tanbouly DM, Zaki HF (2018). Topiramate mitigates 3-nitropropionic acid-induced striatal neurotoxicity via modulation of AMPA receptors. Food Chem Toxicol.

[52] Gupta V, Dhull DK, Joshi J, Kaur S, Kumar A (2020). Neuroprotective potential of azilsartan against cerebral ischemic injury: possible involvement of mitochondrial mechanisms. Neurochem Int.

[53] Alzahrani YM, Mai MA, Kamel FO, Ramadan WS, Alzahrani YA (2020). Possible combined effect of perindopril and Azilsartan in an experimental model of dementia in rats. Saudi Pharm J.

[54] Dhir A, Akula KK, Kulkarni SK (2008). Tiagabine, a GABA uptake inhibitor, attenuates 3-nitropropionic acid-induced alterations in various behavioral and biochemical parameters in rats. Prog Neuro-Psychopharmacol Biol Psychiatry.

[55] Schulz JB, Matthews RT, Henshaw DR, Beal MF (1996). Neuroprotective strategies for treatment of lesions produced by mitochondrial toxins: Implications for neurodegenerative diseases. Neuroscience.

[56] Abiodun OA, Ola MS (2020). Role of brain renin angiotensin system in neurodegeneration: an update. Saudi J Biol Sci.

[57] Kangussu LM, Rocha NP, Valadão PAC, Machado TCG, Soares KB, Joviano-Santos JV (2022). Renin-angiotensin system in Huntington′s disease: evidence from animal models and human patients. Int J Mol Sci.

[58] Mattson MP, Camandola S (2001). NF-κB in neuronal plasticity and neurodegenerative disorders. J Clin Invest.

[59] Jang M, Lee MJ, Cho IH (2014). Ethyl pyruvate ameliorates 3-nitropropionic acid-induced striatal toxicity through anti-neuronal cell death and anti-inflammatory mechanisms. Brain Behav Immun.

[60] Suzuki Y, Ruiz-Ortega M, Lorenzo O, Ruperez M, Esteban V, Egido J (2003). Inflammation and angiotensin II. Int J Biochem Cell Biol.

[61] Jang M, Lee MJ, Kim CS, Cho IH (2013). Korean red ginseng extract attenuates 3-nitropropionic acid-induced Huntington’s-like symptoms. Evidence-Based Complement Altern Med.

[62] Khoshnan A, Ko J, Watkin EE, Paige LA, Reinhart PH, Patterson PH (2004). Activation of the IκB kinase complex and nuclear factor-κB contributes to mutant huntingtin neurotoxicity. J Neurosci.

[63] Sivandzade F, Prasad S, Bhalerao A, Cucullo L (2019). NRF2 and NF-қB interplay in cerebrovascular and neurodegenerative disorders: molecular mechanisms and possible therapeutic approaches. Redox Biol.

[64] Coward WR, Okayama Y, Sagara H, Wilson SJ, Holgate ST, Church MK (2002). NF-kappa B and TNF-alpha: a positive autocrine loop in human lung mast cells?. J Immunol.

[65] Nam J, Aguda BD, Rath B, Agarwal S (2009). Biomechanical thresholds regulate inflammation through the NF-kappaB pathway: experiments and modeling. PLoS ONE.

[66] Saroj P, Bansal Y, Singh R, Akhtar A, Sodhi RK, Bishnoi M (2021). Neuroprotective effects of roflumilast against quinolinic acid-induced rat model of Huntington’s disease through inhibition of NF-κB mediated neuroinflammatory markers and activation of cAMP/CREB/BDNF signaling pathway. Inflammopharmacology.

[67] Sayed RH, Ghazy AH, Yammany MFE (2022). Recombinant human erythropoietin and interferon-β-1b protect against 3-nitropropionic acid-induced neurotoxicity in rats: possible role of JAK/STAT signaling pathway. Inflammopharmacology.

[68] Nogawa S, Zhang F, Elizabeth Ross M, Iadecola C (1997). Cyclo-oxygenase-2 gene expression in neurons contributes to ischemic brain damage. J Neurosci.

[69] Xiang Z, Ho L, Valdellon J, Borchelt D, Kelley K, Spielman L (2002). Cyclooxygenase (COX)-2 and cell cycle activity in a transgenic mouse model of Alzheimer’s disease neuropathology. Neurobiol Aging.

[70] Gopinath K, Sudhandiran G (2012). Naringin modulates oxidative stress and inflammation in 3-nitropropionic acid-induced neurodegeneration through the activation of nuclear factor-erythroid 2-related factor-2 signalling pathway. Neuroscience.

[71] Chen H, Han C, Ren L, Wang F, Cui J, Ha C (2022). The protective effects of Azilsartan against hypoxia in endometrial stromal cells: an implication in endometriosis. Arch Med Sci.

[72] Kumar H, Koppula S, Kim I-S, Vasant More S, Kim B-W, Choi D-K (2012). Nuclear factor erythroid 2-related factor 2 signaling in Parkinson disease: a promising multi therapeutic target against oxidative stress, neuroinflammation and cell death. CNS Neurol Disord Drug Targets.

[73] Napolitano M, Zei D, Centonze D, Palermo R, Bernardi G, Vacca A (2008). NF-kB/NOS cross-talk induced by mitochondrial complex II inhibition: implications for Huntington’s disease. Neurosci Lett.

[74] Kim SF, Huri DA, Snyder SH (2005). Medicine: inducible nitric oxide synthase binds, S-nitrosylates, and activates cyclooxygenase-2. Science.

[75] Ibrahim WW, Abdel Rasheed NO (2022). Diapocynin neuroprotective effects in 3-nitropropionic acid Huntington’s disease model in rats: emphasis on Sirt1/Nrf2 signaling pathway. Inflammopharmacology.

[76] Liu SJ, Liu XY, Li JH, Guo J, Li F, Gui Y (2018). Gastrodin attenuates microglia activation through renin-angiotensin system and Sirtuin3 pathway. Neurochem Int.

[77] Liot G, Bossy B, Lubitz S, Kushnareva Y, Sejbuk N, Bossy-Wetzel E (2009). Complex II inhibition by 3-NP causes mitochondrial fragmentation and neuronal cell death via an NMDA- and ROS-dependent pathway. Cell Death Differ.

[78] Shawki SM, Saad MA, Rahmo RM, Wadie W, El-Abhar HS (2021). Liraglutide improves cognitive and neuronal function in 3-NP rat model of Huntington’s disease. Front Pharmacol.

[79] Wang T, Zhang L, Jiang L, He N (2008). Neurotoxicological effects of 3-nitropropionic acid on the neonatal rat. Neurotoxicology.

[80] Ahmed LA, Darwish HA, Abdelsalam RM, Amin HAA (2016). Role of rho kinase inhibition in the protective effect of Fasudil and simvastatin against 3-nitropropionic acid-induced striatal neurodegeneration and mitochondrial dysfunction in rats. Mol Neurobiol.

[81] Prajapati P, Kumar A, Singh J, Saraf SA, Kushwaha S (2023). Azilsartan ameliorates skeletal muscle wasting in high fat diet (HFD)-induced sarcopenic obesity in rats via activating Akt signalling pathway. Arch Gerontol Geriatr.

[82] Hama Amin RR, Aziz TA (2022). Gastroprotective effect of azilsartan through ameliorating oxidative stress, inflammation, and restoring hydroxyproline, and gastrin levels in ethanol-induced gastric ulcer. J Inflamm Res.

[83] Gupta V, Dhull DK, Joshi J, Kaur S, Kumar A (2020). Neuroprotective potential of azilsartan against cerebral ischemic injury: possible involvement of mitochondrial mechanisms. Neurochem Int.

[84] Colín-González AL, Luna-López A, Königsberg M, Ali SF, Pedraza-Chaverrí J, Santamaría A (2014). Early modulation of the transcription factor Nrf2 in rodent striatal slices by quinolinic acid, a toxic metabolite of the kynurenine pathway. Neuroscience.

[85] Maher J, Yamamoto M (2010). The rise of antioxidant signaling—the evolution and hormetic actions of Nrf2. Toxicol Appl Pharmacol.

[86] Buelna-Chontal M, Zazueta C (2013). Redox activation of Nrf2 & NF-κB: a double end sword?. Cell Signal.

[87] Stack C, Ho D, Wille E, Calingasan NY, Williams C, Liby K (2010). Triterpenoids CDDO-ethyl amide and CDDO-trifluoroethyl amide improve the behavioral phenotype and brain pathology in a transgenic mouse model of Huntington’s disease. Free Radic Biol Med.

[88] Calkins MJ, Jakel RJ, Johnson DA, Chan K, Yuen WK, Johnson JA (2005). Protection from mitochondrial complex II inhibition in vitro and in vivo by Nrf2-mediated transcription. Proc Natl Acad Sci U S A.

[89] Zhang C, Zhao Y, Yang X (2022). Azilsartan attenuates lipopolysaccharide-induced acute lung injury via the Nrf2/HO-1 signaling pathway. Immunol Res.

[90] Wardyn JD, Ponsford AH, Sanderson CM (2015). Dissecting molecular cross-talk between Nrf2 and NF-κB response pathways. Biochem Soc Trans.

[91] Innamorato NG, Rojo AI, García-Yagüe ÁJ, Yamamoto M, de Ceballos ML, Cuadrado A (2008). The transcription factor Nrf2 is a therapeutic target against brain inflammation. J Immunol.

[92] Liu GH, Qu J, Shen X (2008). NF-κB/p65 antagonizes Nrf2-ARE pathway by depriving CBP from Nrf2 and facilitating recruitment of HDAC3 to MafK. Biochim Biophys Acta Mol Cell Res.

[93] Dedeoglu A, Ferrante RJ, Andreassen OA, Dillmann WH, Beal MF (2002). Mice overexpressing 70-kDa heat shock protein show increased resistance to malonate and 3-nitropropionic acid. Exp Neurol.

